# Limb Girdle Muscular Dystrophy Type 2B (LGMD2B): Diagnosis and Therapeutic Possibilities

**DOI:** 10.3390/ijms25115572

**Published:** 2024-05-21

**Authors:** Bal Hari Poudel, Sue Fletcher, Steve D. Wilton, May Aung-Htut

**Affiliations:** 1 Centre for Molecular Medicine and Innovative Therapeutics, Health Futures Institute, Murdoch University, Perth, WA 6150, Australia; bal.poudel@murdoch.edu.au (B.H.P.); s.fletcher@murdoch.edu.au (S.F.); s.wilton@murdoch.edu.au (S.D.W.); 2 Perron Institute for Neurological and Translational Science, The University of Western Australia, Perth, WA 6009, Australia; 3 Central Department of Biotechnology, Tribhuvan University, Kirtipur, Kathmandu 44618, Nepal

**Keywords:** dysferlinopathies, LGMD2B, antisense oligonucleotides, readthrough therapy, gene therapy

## Abstract

Dysferlin is a large transmembrane protein involved in critical cellular processes including membrane repair and vesicle fusion. Mutations in the dysferlin gene (*DYSF*) can result in rare forms of muscular dystrophy; Miyoshi myopathy; limb girdle muscular dystrophy type 2B (LGMD2B); and distal myopathy. These conditions are collectively known as dysferlinopathies and are caused by more than 600 mutations that have been identified across the *DYSF* gene to date. In this review, we discuss the key molecular and clinical features of LGMD2B, the causative gene *DYSF*, and the associated dysferlin protein structure. We also provide an update on current approaches to LGMD2B diagnosis and advances in drug development, including splice switching antisense oligonucleotides. We give a brief update on clinical trials involving adeno-associated viral gene therapy and the current progress on CRISPR/Cas9 mediated therapy for LGMD2B, and then conclude by discussing the prospects of antisense oligomer-based intervention to treat selected mutations causing dysferlinopathies.

## 1. Introduction

Dysferlinopathies are a family of autosomal recessive muscular disorders caused by mutations in the dysferlin gene (*DYSF)* and include the following diseases: Limb-girdle muscular dystrophy type 2B (LGMD2B; MIM #253601), Miyoshi myopathy (MM; MIM #254130), and distal myopathy anterior tibial onset (DMAT; OMIM #606768). Dysferlin (*DYSF*) is primarily expressed in skeletal and cardiac muscles, where it encodes a ~237 kDa plasma membrane protein that plays a crucial role in the repair of damaged muscle cell membranes [1,2]. The protein encoded by dysferlin belongs to the ferlin protein family that is characterized by the presence of a type II transmembrane domain, with the majority of the domain adjacent to the cytoplasm, as well as multiple calcium binding (C2) domains that are involved in calcium-dependent membrane fusion events [3,4]. As with most muscular dystrophies, there is currently no curative treatment available for dysferlinopathies. However, preliminary in vitro and in vivo studies using viral gene therapy, CRISPR/CaS9, and antisense oligonucleotide (AO) technologies have shown promise in treating limb girdle muscular dystrophy 2B (LGMD2B). Among these, AO-mediated splice switching presents a promising strategy to treat some mutations causing LGMD2B. Antisense oligonucleotide-mediated intervention during pre-mRNA processing has been clinically approved for selected mutations causing Duchenne muscular dystrophy, reactivating inappropriately processed gene transcripts as a treatment for spinal muscular atrophy and a single patient with Batten’s disease [5,6].

### 1.1. Dysferlin

The dysferlin gene (*DYSF*) is located on chromosome 2p12-14 and composed of 55 exons, spread over 150 Kb of genomic DNA, and encodes a protein of ~237 kDa. As mentioned previously, defects in *DYSF* can manifest as different types of muscular dystrophy based on clinical presentation [7,8]. The weakening and degeneration of muscles in the pelvic and shoulder girdles, often accompanied by a deficiency or absence of the dysferlin protein, typically exhibit in one’s third or fourth decade of life. Nonetheless, in severe instances, this reduction or absence of dysferlin may become evident earlier [9].

The contraction of voluntary skeletal muscles generates the energy required for both motility and strength. As such, skeletal muscle cells are subjected to significant contractile forces and stresses, frequently resulting in disruption of the muscle surface membrane (sarcolemma). Dysferlin assists in repairing the disrupted membrane in association with Ca^+2^ binding proteins annexins (A1, A2 and A6), mitsugumin, and S100A [10]. As a consequence of insufficient amounts of, or non-functional, dysferlin, the accumulation of membrane damage eventually leads to muscular dystrophy.

The *DYSF* promoter region contains two CpG islands, a TATA box, and two clusters of binding sites for various transcription factors, including those involved in muscle-specific dysferlin expression. Alternative splicing of exons 5, 17, and 40 has been previously reported [11]. Using 5′ rapid amplification of the cDNA ends on the adult skeletal muscle total RNA, Pramono et al. (2006) identified a splice variant of *DYSF*, *DYSF-V1* (Ref seq NM_003494.4), that arises from alternate exon inclusion in the 5′ UTR [12]. Pramono and colleagues had previously reported other novel transcripts due to the inclusion of exons derived from either intron 5 or 40 [12]. An alternative transcript generated through omission of exon 17 was also reported [12]. High levels of *DYSF* mRNA expression have been reported in blood, skeletal muscle, heart, and the placenta, with lower expression observed in the brain, kidney, lung, and even less in the liver and pancreas. According to Aoki et al. (2001), the largest reported dysferlin cDNA sequence spans 6.9 Kb and is found in skeletal muscle [13], whereas in the brain, the major transcript reported has a length of only 3.8 Kb, with the highest expression being reported in the putamen and essentially no expression in the spinal cord and fetal brain [14,15].

**Figure 1:**
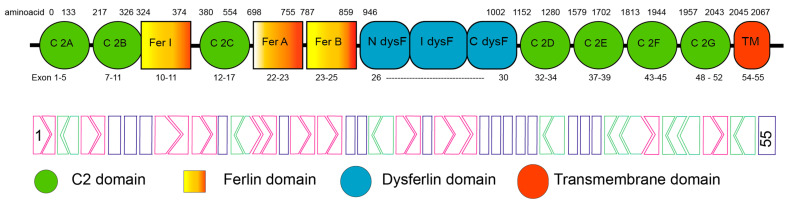
Architecture of the human dysferlin protein. C2A–C2G; Calcium-binding C2 domains, TM; transmembrane domain, Fer; Ferlin and dysF; dysferlin domains. The amino acid residue positions are indicated above the domain structure, with the encoding exons below. The dysferlin exon map with a reading frame is shown below the domain structure, with the rectangular blocks indicating exons with splice junctions occurring between codons, whereas exons with chevron sides indicate splice junctions that occur within a codon <1:2< or >2:1>. Adapted from Abdullah et al., 2014 [17], Sula et al., 2014 [20], and NM_003494.4.

The cytoplasmic region of dysferlin contains seven calcium-binding domains (C2A to C2G, from N-terminus to C-terminus) that are thought to mediate vesicle fusion with the plasma membrane. In addition, dysferlin has multiple domains including ferlin “fer” and dysferlin “dysF” domains. Various calcium-dependent lipid-binding C2 (C2), “fer”, and “dysF” domains are shown in Figure 1 [4]. Ferlin domain A (FerA) of dysferlin binds to phospholipids and plays an integral role in membrane fusion activity in a calcium-dependent manner [16]. The C2 domains are independently folded blocks of approximately 130 residues that assemble into a beta-sandwich motif containing eight anti-parallel beta-sheets [4]. These C2 domains also participate in lipid and protein binding [17] and are generally involved in membrane interactions or fusion events, or in the generation of secondary messenger lipids involved in signal transduction pathways [18,19].

### 1.2. The Role of Dysferlin in Membrane Repair and the Intracellular Vesicular System

In muscle cells, dystrophin–glycoprotein complex (DGC) proteins maintain muscle membrane integrity and structure, while the muscle membrane repair complex rapidly repairs sarcolemmal tears and ruptures. Dysferlin is one of the most important proteins in the sarcolemmal repair process, although deficiencies of any protein in this repair complex would lead to muscular dystrophy, a heterogeneous group of muscle-wasting diseases [4].

According to the membrane repair model for dysferlin, damage to the membrane influences the diffusion of calcium within the muscle fibers, resulting in a zone of high calcium around the disrupted site [21]. In the presence of localized high levels of calcium, dysferlin-carrying repair vesicles are directed to the site of damage, where they accumulate and fuse with one another and the plasma membrane. Dysferlin then facilitates vesicle docking and fusion with the plasma membrane by interacting with annexin A2, other dysferlin molecules, and additional unknown protein-binding molecules [2,21,22]. Fusion of the repair vesicles with the plasma membrane acts as a “patch” across the disruption and thereby reseals the damaged plasma membrane [10,21]. Research into other pathways associated with dysferlin-linked membrane repair is still ongoing.

**Figure 2:**
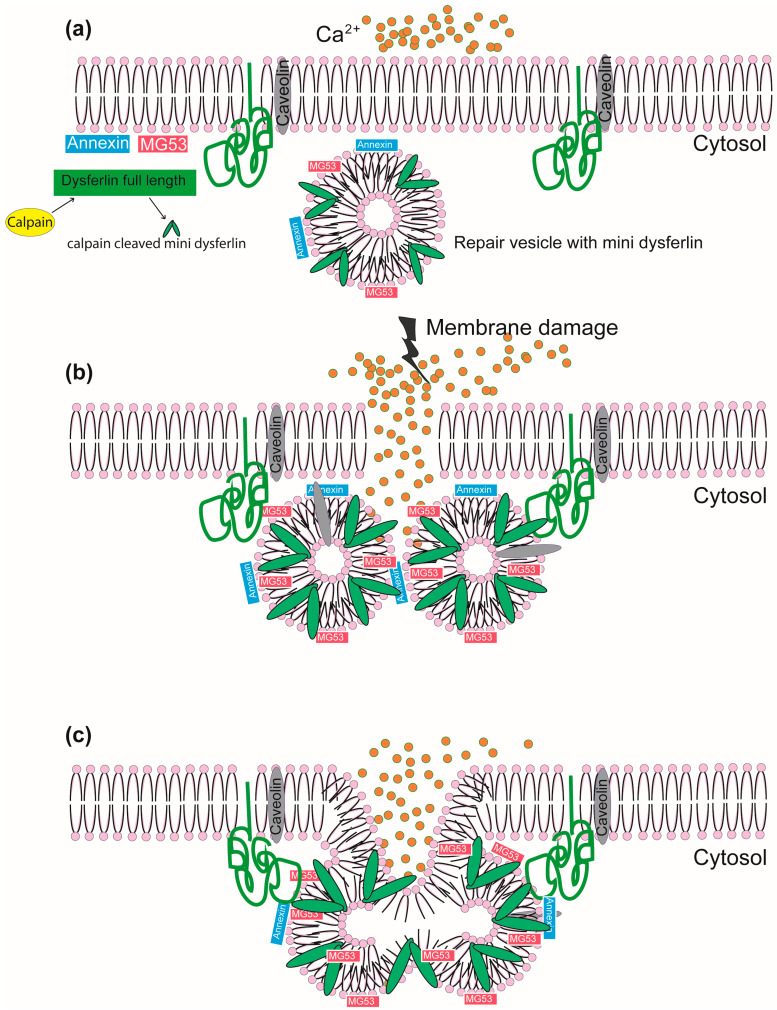
The role of dysferlin in membrane repair in a calcium-dependent manner. (**a**) Normal sarcolemmal membrane showing the location of dysferlin (green), annexin (blue), MG53 (pink), and caveolin (grey). (**b**) Membrane damage causes high influx of calcium into the cytosol and membrane repair vesicles loaded with mini-dysferlin (dysferlin are cleaved by calpain to form mini-dysferlin) are brought near the site of injury by the interaction of multiple proteins as shown. (**c**) The membrane is repaired by sealing the lesion by patch formation. Modified from Renzhi Han 2011 and Lek et al., 2013 [24,25].

Dysferlin also interacts with other proteins to repair membrane degeneration. Sharma et al. (2010) indicated a novel dysferlin interacting partner—platelet endothelial cellular adhesion molecule-1 (PECAM-1). PECAM-1 is an essential adhesion molecule for angiogenesis regulation, which adds to our understanding of the functional roles of ferlins in angiogenesis and selective protein trafficking in a vascular setting [23]. Lek et al. (2013) described the interaction of dysferlin with a previously uncharacterized membrane repair protein, mitsugumin-53 (MG53), an E3 ubiquitin ligase that is rapidly recruited to injury sites [24]. They showed that an injury-specific calpain cleaves dysferlin into a ~72 kDa C-terminal dysferlin isoform, a mini-dysferlin_C72_ that is recruited to the sites of membrane injury rather than the full-length dysferlin. They reported that mini-dysferlin_C72_-rich vesicles are rapidly recruited to injury sites and fuse with plasma membrane compartments decorated by MG53 in a process coordinated by L-type calcium channels [24]. The role of dysferlin in the repair of a damaged sarcolemma membrane is shown in Figure 2.

Dysferlin also plays an important role in regulating calcium (Ca^2+^) signaling by maintaining the balance of Ca^2+^ levels in T-tubule membranes, particularly around the triad junction [26,27]. Muriel et al. conducted a study investigating the function of each dysferlin C2 domain [28]. They found that specific domains are involved in Ca^2+^ signaling, while others contribute independently to the process of membrane repair. Additionally, they observed that the absence of dysferlin leads to a reduction in the strength of the voltage-induced Ca^2+^ signals. They suggested that dysferlin may aid in optimizing the interaction between the L-type calcium channel (LTCC) and the ryanodine receptor (RyR1) at the triad junction [28]. Furthermore, Wang et al. demonstrated that the dysferlin C2A domain binds with two calcium ions, resulting in a more stable structure that enhances calcium binding [29].

Mitochondria, cellular energy generators that produce adenosine triphosphate (ATP) by oxidative phosphorylation, are also involved in Ca^2+^ buffering. Vincent et al. (2016) hypothesized that mitochondrial defects may be evident in the skeletal muscle biopsies from dysferlinopathy patients. Their analyses revealed that the percentage of mitochondrial complex I- and complex IV (cytochrome c oxidase)-deficient muscle fibers was higher in patients with *DYSF* mutations than in the healthy controls [30]. However, it was noted that these patients did not exhibit any rearrangements in their mitochondrial DNA.

Furthermore, the membrane repair function of dysferlin has been linked to the soluble N-ethylmaleimide-sensitive factor (SNARE). The direct interaction between dysferlin and the SNARE proteins, syntaxin 4 and SNAP-23, was reported by Codding et al. (2016). In addition, dysferlin accelerated the syntaxin4/SNAP23 complex formation and SNARE mediated lipid mixing in a calcium-dependent manner [31].

Through interactions with other proteins, dysferlin also contributes to tubule formation and many studies have provided additional evidence for this function [32,33]. Demonbreun et al. (2014) showed that transverse tubule formation and glycerol sensitivity were regulated through dysferlin and myoferlin [34]. The lack of functional ferlins and reduced vesicle trafficking causes the accumulation of lipids. In the absence of functional ferlins, T-tubules and plasma membranes are unable to repair damage. A leaky sarcolemma results in the release of lipids, including glycerol, which promotes myopathy by functionally separating the T-tubules from the sarcolemma and sarcoplasmic reticulum [35]. Ultimately, adipogenesis and the accumulation of adipocytes are promoted within the muscle, which leads to an increased production of glycerol, a by-product of lipolysis [36]. Furthermore, dysferlin assists with the arrangement of liposomes, and produces a T-tubule-like membrane system in non-muscle cells that facilitates the biogenesis of the T-tubules system [32]. Finally, dysferlin has also been reported to interact with the proteins associated with human neuroblast differentiation, such as AHNAK, affixin, S100A10 [37], calpain, and dihydropyridine receptor [38]. In summary, dysferlin is a protein crucial for the maintenance of membrane repair; hence, reduced dysferlin expression or the production of non-functional dysferlin protein isoforms due to null or missense mutations can cause muscle-wasting diseases.

### 1.3. Mutations in Dysferlin and LGMD2B

Liu et al. (1998) identified *DYSF* mutations in patients with dysferlinopathies and observed that the same *DYSF* mutations can present more than one disease phenotype in different individuals [1]. Sinnreich et al. (2006) identified a single base substitution in a highly conserved branch point sequence of intron 31 that led to the exclusion of exon 32 from the mature mRNA [39]. Western blot analysis revealed a reduction in dysferlin levels to about 10% of those observed in healthy controls, contributing to a mild phenotype. This observation indicated that a proportion of the transcript may have escaped the exon 32 exclusion, producing some dysferlin protein. Mutation studies by Mafalda et al. (2011) confirmed the primary involvement of *DYSF* in the LGMD 2B/MM phenotypes [40], providing the first direct and conclusive evidence that dysferlin protein levels lower than 10% of those in healthy individuals are considered pathogenic [40].

**Figure 3:**
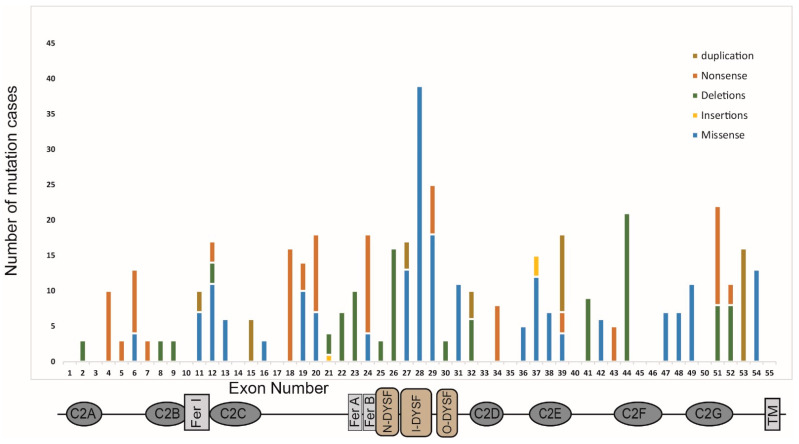
A schematic showing the protein structure of dysferlin and the mutation spectrum. The positions of each of the seven C2 domains (C2A–C2G), the FerA/B, *DYSF*, and transmembrane domains, and the exon arrangement are shown on the *y*-axis. The histogram represents the incidence of pathogenic mutations within each of the 55 exons encoding the canonical skeletal muscle isoform of dysferlin (data derived from the Leiden muscular dystrophy database) [8].

Due to the constantly increasing spectrum of *DYSF* mutations, the Universal Mutation Database for Dysferlin (UMD-DYSF) was established to manage the expanding mutational dataset [41]. UMD-DYSF is a locus-specific database that contains extensive data related to *DYSF* mutations [8,41] and provides a comprehensive account of the *DYSF* disease-causing mutations reported in the literature [8]. According to the UMD-DYSF, different types of mutations, which include nonsense, missense, and indels, are dispersed relatively evenly throughout *DYSF*, as shown in Figure 3. Missense mutations are the most abundant followed by deletion and nonsense mutations (Figure 3) and insertion is the least reported. In addition to the recurrent mutations, the UMD-DYSF database also lists seven founder mutations [41]. The Portuguese population shows two founder mutations c.1180_1180+7delAGTGCGTG (r.1054_1284del, p.Glu353_Leu429del) and c.5492G>A. Founder mutations in the Canadian (c.2372C>G (p.Pro791Arg)), Italian (c.2875C>T (p.Arg959Trp), Caucasian Jewish c.2779delG (p.Ala927LeufsX21), and Lebanese Jewish (c.4872_4876delinsCCCC (p.Glu1624AspfsX9)) populations are reported. Lastly, 2% of Spanish dysferlinopathy patients from the region of Sueca have a c.5713C>T (p.Arg1905X) founder mutation. Izumi et al. (2020) reported a c.29997G>T; p.Trp999Cys mutation as the most frequent (22.9%) in a Japanese cohort of 209 cases [42]. Similarly, they also found that the frequency of missense mutations was higher (70.6%) in the inner dysferlin domain [42]. However, in a French dysferlinopathy cohort, the incidence of nonsense mutations was found to be higher [43]. The frequency of missense, nonsense, and indel mutations appears to vary across diverse populations as evidenced by several studies on different cohorts [41,44,45].

A *DYSF* mutation study conducted on 245 dysferlinopathy patients of Chinese ethnicity by Zhong et al. (2021) identified 40 novel mutations and observed c.1375dupin 6.5% of the patients [44]. Genomic deletions of exons 3, 33, 34, 35, 40, and 41, and 42 were reported in the same cohort. Yi-Ying Hu and colleagues (2018) reported cases where two different mutations were found (compound heterozygous mutations) in a patient, including a de novo mutation c.613C>T in exon 6 and a novel missense mutation c.968T>C in exon 11 [46].

To further study the consequences of different mutations, disease phenotype, muscle pathology and the effect of drugs, researchers have developed several dysferlin-deficient mouse models, as well as studied naturally occurring dystrophic mouse strains.

### 1.4. Mouse Models to Study Dysferlinopathies

In recent years, several mouse dysferlinopathy models have been identified or developed, including SJL/J, A/J, BLA/J, and Dysf-knock out mice [47,48]. SJL/J mice possess a 171 bp genomic deletion affecting the 3′ splice site of exon 45 [49] in the *DYSF* gene, leading to decreased dysferlin protein levels compared to wild-type mice. Consequently, this alteration results in the spontaneous development of myopathy. The A/J mouse strain is another naturally occurring dysferlin-deficient animal arising from a retrotransposon insertion in *DYSF* intron 4, while BLA/J mice were derived from the SJL/L strain [50]. Despite a compromised dysferlin, the SJL/J and A/J murine models do not exhibit significant muscle weakness. This is not unusual, as some other mouse muscular dystrophy models such as the *mdx* mouse that carries a nonsense mutation in the dystrophin gene do not show obvious symptoms until the animals are of advanced age [51]. Therefore, the screening and analysis of any dysferlin therapies are normally dependent on histopathological examinations, such as observing the fiber shape and size, central nucleation, fibrosis, and inflammation.

To study the consequences of one particular missense mutation, Malcher et al. (2018) developed a new mouse model called MMex38, which carries a missense mutation in *DYSF* exon 38. Similar to the human *DYSF* variant p.Leu1341Pro, this particular variant induces all the characteristics of a dysferlinopathy arising from a missense mutation, including progressive muscular dystrophy, amyloid formation, and defects in membrane repair [52]. The MMex38 mouse model was employed for a study where U7 small nuclear RNA (snRNA)-mediated splice switching was used to induce dual exon 37 and 38 skipping from the *DYSF* transcript. Dysferlin exon skipping was assessed in vitro in C2C12 murine cells with a reported efficiency of 12.9%. The subsequent in vivo study carried out in MMex38 mice showed less efficient exon skipping as determined by RT-PCR, but an increase in dysferlin protein functionality was reported [52].

#### Stem Cells as a Model

Patient-derived induced pluripotent stem cells (iPSCs) have been used as an in vitro model to test potential drug compounds. Kokubu et al. (2019) screened small molecules using iPSCs generated from a patient with compound heterozygous mutations; a missense mutation c.2997G>T(p.W999C) and a nonsense mutation c.1958delG, and observed that nocodazole was found to increase the level of misfolded dysferlin expression in cells with increased membrane resealing following injury by irradiation when compared to healthy controls [53]. Further research is required to assess the effectiveness of nocodazole against various pathogenic missense mutations present in dysferlin, as illustrated in Figure 3. Additionally, it is necessary to establish optimized growth and differentiation protocols to enable reliable safety assessments using iPSCs as a cellular model and for cell replacement therapy.

## 2. LGMD2B Disease Symptoms and Diagnosis

Dysferlinopathies are characterized by the atrophy and weakness of the gastrocnemius muscle and/or the anterior tibial muscles, along with elevated creatine kinase (CK). In muscle biopsies, mononuclear cell infiltration may be observed [54,55] and can often be misdiagnosed as an inflammatory myopathy, such as polymyositis. Misdiagnosis can lead to inappropriate treatment with anti-inflammatory drugs such as corticosteroids, that are ineffective in treating the consequences of dysferlin mutations and typically result in many adverse side effects, including loss of muscle strength, reduced bone density, hypertension, cataracts, and diabetes [56].

Mutations affecting the structure and functions of the dysferlin protein manifest with chronic muscle fiber loss, fat replacement, and fibrosis [57]. Clinical symptoms of dysferlinopathies include progressive muscle weakness, increased serum kinase (CK), inflammation, and abnormal muscle morphology. Approximately 30 percent of LGMD2B patients become wheelchair dependent within 15 years from disease onset, but severity and progression can vary significantly between populations [58,59]. Although many studies have been conducted, the exact prevalence of LGMD2B is still not known and can only be estimated [60], which is between 1 in 14,286 and 1 in 200,000 [41]. Others have estimated a smaller prevalence range, from 1 in 14,500 to 1 in 123,000 people, with LGMD2B thought to account for between 3% and 19% of all LGMDs [41].

Immunohistochemistry on muscle biopsy can be inconsistent when using the Novocastra antibodies NCL-Hamlet and NCL-Hamlet-2 to confirm diagnosis [61]. Western blotting is the most proficient method for determining diagnosis based on quantity and molecular size of the 237 kDa protein. In affected subjects, dysferlin levels can vary from partial deficiency to total absence. Matching the phenotype with dysferlin levels has been challenging. Anderson et al. (2000) demonstrated low levels in homozygous frameshifting, none in nonsense/frameshift, mid-range in frameshift, and deletion mutations and high levels in missense mutations [62]. In another study, all reported pathogenic dysferlin mutations affected the protein expression level in skeletal muscle [63].

Genomic analysis of the *DYSF* gene is the most effective approach to detect pathogenic variants [58]. Through whole genome sequencing, changes such as synonymous mutations that influence splicing, splice site mutations, deletions and duplications in coding and noncoding regions can be discovered. Similarly, whole transcriptome sequencing allows for the characterization of all types of transcripts (both coding and noncoding). After confirming the presence of a pathogenic mutation, genetic counseling becomes accessible, marking the initial phase in the implementation of gene/cell replacement or mutation-specific therapies.

## 3. Therapeutic Strategies for Dysferlinopathies

**Figure 4:**
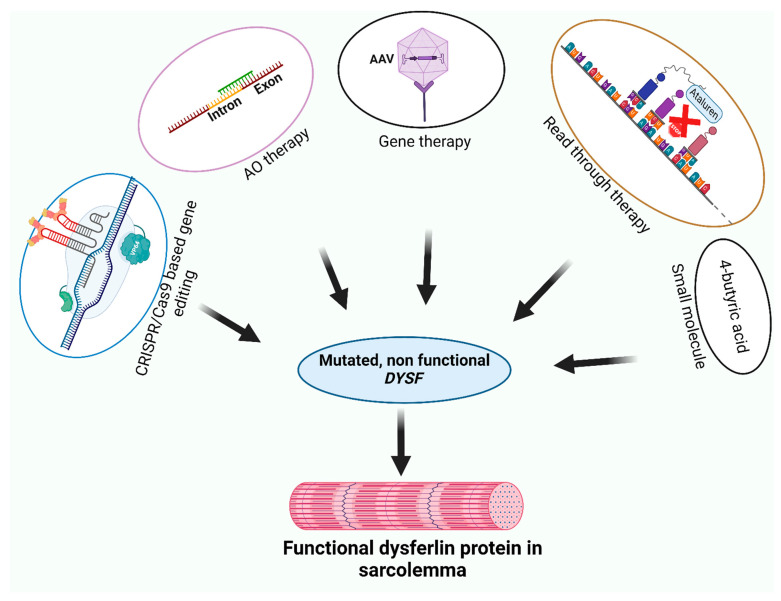
Some possible therapeutic strategies at a glance. CRISPR/Cas9 mediated correction of mutations, antisense oligomer (AO) therapies (exon skipping, exon retention, splice correction), gene therapy, readthrough (red X is blocking of stop codon by Ataluren) and small molecule-based therapies have shown some potential to restore either fully or partially functional dysferlin expression.

Although there are currently no effective drugs available to treat dysferlinopathies, clinical trials are ongoing with an adeno-associated virus-mediated gene replacement strategy (NCT02710500) [64], as well as various preliminary studies using gene and AO therapies in mouse models and patient-derived cells. In the following sections, the current studies and outcomes of adeno-associated viral (AAV) vector-based gene therapy, CRISPR (clustered regularly interspaced short palindromic repeats)/Cas9 gene correction, small molecule and nonsense mutation readthrough approaches, as well as AO interventions are discussed and shown in Figure 4.

### 3.1. Adeno-Associated Virus-Mediated Gene Therapy

Adeno-associated virus vectors infect dividing and non-dividing cells and are extensively used for gene therapy applications; favored for their relatively low immunogenicity, lack of pathogenicity, and ability to establish long-term transgene expression [65]. AAV-mediated gene therapy for dysferlinopathy involves introducing functional copies of the dysferlin gene into affected cells to compensate for the defective or absent dysferlin protein. This can potentially restore normal muscle function and alleviate symptoms associated with dysferlinopathies, such as muscle weakness and degeneration.

One of the major limitations of recombinant AAVs is the limited cargo capacity of 4.7 Kb. The *DYSF*-protein-coding sequence spans 6.9 Kb, thus severely restricting its suitability for AAV-mediated gene transfer therapy, although it has been suggested that larger genomes can be packaged into some AAV vectors generated through rare recombination events [66].

An alternative gene transfer strategy is packaging the first (exons 1–29) and second half (exons 29–55) of the *DYSF* cDNA into two separate AAV vectors. These two dysferlin transcripts contain donor and acceptor splice site sequences to facilitate trans-splicing and generate a full-length dysferlin coding transcript. A preclinical study conducted by Lostal et al. (2010) demonstrated that intravenous injection of viral constructs into dysferlin-deficient mice resulted in the expression of full-length dysferlin and led to an improvement in function compared to untreated or placebo-treated controls [67]. Dysferlin function was assessed histologically by a reduction in the number of necrotic fibers, restoration of membrane repair capacity in the muscle, and a global improvement in locomotor activity [67]. A similar preclinical study was performed by Sondergaard and colleagues who used the dual-vector system to package the *DYSF* cDNA into AAV serotype rh.74 using two discrete vectors with a 1 Kb region of homology [68]. They showed an increase in dysferlin expression in mice and non-human primates after intramuscular and vascular delivery. Similarly, in a separate in vivo study, Potter et al. (2018) delivered full-length 6.9 Kb dysferlin cDNA using dual AAV vectors. Dysferlin-deficient mice were injected with the AAV viral constructs systemically and an improvement in membrane repair, comparable to that of wild-type levels, was reported [69]. An AAV construct (rAAVrh74.MHCK7.DYSF.DV) developed by Sarepta Therapeutics is another dual vector system under clinical evaluation in a phase I safety and tolerability study. This AAV construct rAAVrh74.MHCK7.DYSF.DV is a dual recombinant AAV carrying each half of the dysferlin transgene under control of the muscle- and heart-specific MHCK7 enhancer [70,71] (NCT02710500).

A separate clinical trial has assessed the safety, effectiveness, and tolerability of SRP-6004, a dual-vector AAV gene therapy, when delivered intravenously to ambulatory patients (NCT05906251) [72]. Adaptive and innate immune responses are major limitations of viral vector-mediated gene therapies [73]. A number of clinical trials using AAVs are on hold due to serious adverse events, including deaths from 2020 to 2021 [74,75,76]. Astellas reported the deaths of four participants enrolled in their AT132 gene therapy trial for X-linked myotubular myopathy, which was halted twice due to safety concerns [75]. The deaths of three participants were linked to the high dosages; however, the cause of the fourth, not related to high dosage, is unknown. Similarly, Biomarin’s BMN 307 gene therapy drug for phenylketonuria has been placed on hold after liver tumors were observed in the mice administered with BMN 307 [75].

### 3.2. CRISPR/Cas9-Mediated Precise Correction for Pathogenic DYSF Mutations

CRISPR/Cas9 is an antiviral mechanism present in some bacteria that has become an exciting and popular tool for gene editing [77,78]. The CRISPR/Cas9 system relies on the Cas9 nuclease to be directed to specifically bind and cleave a nucleic acid sequence through the annealing of the guide RNA sequence (gRNA sequence). After cleavage, the DNA can be edited, replaced, or re-joined by nonhomologous end joining (NHEJ) or through homology-directed repair (HDR) mechanisms [79]. There are two classes of CRISPR/Cas systems (class I and class II) present in different bacteria, classified according to the structure and functions of the Cas protein. Class I includes type I, III, and IV, and class II includes type II, V, and VI, based on the target. DNA is recognized and cleaved by Type I, II, and V systems, whereas type VI edits RNAs and type III can edit both RNA and DNA. Turan et al. (2016) used CRISPR/Cas9 for the precise in vitro correction of disease-causing mutations in iPSCs derived from patients with LGMD2B [80,81]. They were able to correct a dysferlin nonsense mutation, c. 5713C>T; p.R1905X and the most common alpha-sarcoglycan mutation, a missense mutation, c.229C>T; p. R77C, using the CRISPR/Cas9 gene editing system [81]. Although the authors claimed this technique was promising, the efficiency of allele-specific correction was only 0.7–1.5%, and off-target effects were also observed, another major limitation that needs to be addressed and overcome.

### 3.3. Readthrough of Nonsense Mutations to Treat Dysferlinopathies

Bypassing nonsense mutations with some compounds that influence recognition of premature termination codons could generate functional dysferlin protein. However, this readthrough approach can be applied only to nonsense mutations, which account for ~25% of recurrent mutations in dysferlinopathy, as shown in Figure 3. Some antibiotics, in particular aminoglycosides such as gentamicin can induce readthrough of nonsense mutations. Gentamicin was tested in Duchenne muscular dystrophy (DMD) patients and dystrophin protein was induced; however, there were severe side effects such as nephrotoxicity and ototoxicity which precluded gentamicin for long-term usage [82]. Ataluren (PTC124) is another small molecule reported to bypass premature stop codons in DMD [83] and other disorders, like Leber congenital amaurosis type 4 (LCA4) [84], cystic fibrosis transmembrane conductance regulator (CFTR) [85], and Usher Syndrome 2A [86]. It was developed by PTC Therapeutics Inc (South Plainfield, NJ) and is currently undergoing phase 3 clinical trials for DMD (NCT03179631) [83,87].

Seo et al., 2021 demonstrated that a premature stop codon in a humanized *DYSF* knock-in mouse model (*dqx)* could respond to ataluren treatment, whereby readthrough of the p.Q832* mutation was reported to induce some functional recovery. The *dqx* mouse lacks dysferlin in skeletal muscle from birth but after two weeks of oral ataluren treatment at 0.9 mg/mL, some restoration of dysferlin expression and reduced skeletal muscle pathology was evident. As a negative control, the same treatments in the A/J mouse did not show any improvement, since this animal carries a unique ETn retrotransposon inserted in intron 4 that cannot respond to ataluren treatment [88]. These outcomes support potential treatment of *DYSF* nonsense mutations, providing hope for dysferlinopathy patients carrying a nonsense mutation [88].

### 3.4. Small Molecule Restoration of Membrane Repair Function

4-phenylbutyric acid (4-PBA) can partially restore the membrane repair ability of some mutant dysferlins. Tominaga et al. (2022) demonstrated that 4-PBA can partially restore the membrane localization of 25 different dysferlin missense variants in an HEK cell assay [89]. Subsequently, they showed the rescue of membrane repair ability of the variants in patient-derived cells after treatment with 4-PBA. A two-day oral administration of 4-PBA solution (2 mg/mL in sterile drinking water) in MMex38 mice resulted in expression and localization of dysferlin similar to the untreated, age-matched wild-type animals, whereas the untreated MMex38 mice were membrane-repair deficient [89].

### 3.5. Antisense Oligonucleotide-Mediated Therapies

Antisense oligonucleotides are short, synthetic single-stranded oligonucleotides that can be designed to bind to specific regions of mRNA or pre-mRNA to modify gene expression through several distinct mechanisms, as determined by the AO chemistry [5,90] and target sequence. DNA analogue antisense sequences can activate RNaseH degradation of the target RNA and hence downregulate specific gene expression [91], whereas RNA-like analogues do not induce RNaseH activity and can be used as steric blockers to influence translation or modulate pre-mRNA splicing in order to correct aberrant splicing [92], remove specific exons [93], or retain selected exons as required. Due to rapid degradation by endogenous nucleases, natural DNA and RNA oligonucleotides have limited potential as therapeutic agents. To overcome this limitation, oligonucleotides with modified bases and backbones have been developed to improve stability, enhance binding affinity, and minimize the immune-stimulatory response [94,95].

The splice-switching drug *Exondys 51* was approved by the US Food and Drug Administration in September 2016 for the treatment of the most common subset of DMD-causing dystrophin deletions [96,97,98]. *Exondys 51* is a phosphorodiamidate morpholino oligomer and reframes the dystrophin transcripts disrupted by some deletions flanking DMD exon 51. Restoration of the reading frame generates internally truncated but partially functional protein isoforms, as found in some mildly affected Becker muscular dystrophy (BMD) patients [96]. The more recent approval of additional splice-modulating AO drugs [99], *Golodirsen*, *Viltolarsen* [100] and *Casimersen* [101], to treat other DMD deletions and the approval of *Nusinersen* to treat spinal muscular atrophy has expanded the repertoire of antisense drugs to treat rare diseases. *Nusinersen* is another example of an AO drug that specifically targets a region of the *SMN2* pre-mRNA downstream of exon 7 [102,103]. By binding to a strong silencer element in this pre-mRNA, *Nusinersen* alters the splicing process by promoting the inclusion of exon 7 in the mature *SMN2* mRNA and the production of functional SMN protein [104].

While the exon skipping or exon retention drugs were developed to treat these common rare diseases, perhaps a more remarkable outcome was seen with the approval of the personalized splice-switching AO, *Milasen*, in 2018. The drug was developed to treat a six-year-old child, Mila, who was suffering from Batten’s disease arising from mutations in her neuronal ceroid lipofuscinosis 7 (*CLN7*) gene. Mutational analysis showed a genomic insertion of an SVA(SINE-VNTR-*Alu*) retrotransposon-a DNA sequence that can replicate and insert itself into different parts of the genome. SVAs are composite elements composed of three main parts, namely the SINE (Short Interspersed Nuclear Element), VNTR (Variable Number Tandem Repeat), and Alu (another type of retrotransposon) [105]. They can influence gene expression and may cause genetic mutations or alterations when inserted into genes or regulatory regions of the genome. The SVA insertion creates a cryptic splice-acceptor site in intron 6 in one of her *CLN7* alleles [92], resulting in the retention of a partial *CLN7* intron 6 sequence in the mature mRNA. *Milasen*, a 22 nucleotide (2′-*O*-methoxyethyl modified nucleotides on a phosphorothioate backbone, 2′ *O*Me-PS) AO targeting the *CLN7* intron 6 cryptic splice site reduced this aberrant splice form and forced the splicing machinery to default to the normal splicing pattern. *Milasen* was the first FDA approved drug customized for the use of just one person and progressed from identification of the disease-causing mutation to patient treatment in less than a year. Similar approaches to treat the same type of splicing defect (cryptic splice site activation and pseudo-exon retention) should be feasible for some rare pathogenic mutations in *DYSF*. Furthermore, some nonsense, missense or frame-shifting indels in redundant *DYSF* exons could be by-passed if the loss of that exon in the mature mRNA does not completely compromise dysferlin function.

#### 3.5.1. Antisense Oligonucleotide Mediated Strategies to Address Dysferlinopathy

Sinnreich et al. described two sisters with severe dystrophic symptoms who both possessed homozygous dysferlin null mutations, {(4872delG) and (G4876C)} [39]. Both parents were identified as heterozygous carriers of these mutations. Their mother carried an additional novel substitution of A to G at position –33 in intron 31 (A344333>G) of *DYSF.* This A to G variation compromised the branch point, resulting in skipping of the in-frame exon 32 from the mature mRNA. The dysferlin protein produced from this latter allele appeared to provide partial compensation for the null mutation, consistent with the mother’s less severe phenotype [39]. This report suggests that *DYSF* exon 32 can be removed from the mature mRNA without seriously compromising dysferlin function. Barthelemy et al. (2015) undertook another in vitro study and induced *DYSF* exon 32 skipping using AOs [106]. They also performed an in vitro functional analysis of dysferlin and reported an increase in functional dysferlin expression [106].

Dominov et al. (2014) identified a novel deep intronic point mutation within intron 44, (c.4886+1249 G>T). This mutation activated a cryptic donor splice site that altered the normal splicing of the *DYSF* mRNA by creating an aberrant transcript that contained an extra 177 nucleotides from intron 44. The additional 59 amino acids encoded within the conserved C2F domain of the dysferlin protein completely disrupted function [107]. To correct this aberrant splicing, three 2′ OMe-PS AOs targeting the acceptor and donor sites of the pseudoexon were designed and used to block the abnormal splicing event. Two AOs targeting the donor site of pseudoexon, after transfection into the patient’s cells, restored synthesis of the full-length *DYSF* mRNA with increased dysferlin expression [107]. Subsequently, Dominov et al. (2019) identified a deep intronic point mutation (c.5668-824 C>T) in intron 50 of *DYSF* in another dysferlinopathy patient. The mutation within intron 50 triggers cryptic splicing, leading to the insertion of an additional 180 bases, which results in the premature termination of protein translation within the *DYSF* C2G domain. As with the other examples, these cryptic splicing scenarios could be suppressed by the splice-switching AOs, and efficacy assessed by transcript analysis and Western blot analysis [108].

Pathogenic mutations in dysferlin are dispersed throughout the gene [8,45], and like many other genes, not all mutations can be addressed with splice-switching AOs and hence precise identification and characterization of mutations is crucial. The development of AOs to induce dystrophin exon skipping was informed by the clear genotype–phenotype correlation, with in-frame genomic deletions typically (but not always) associated with the milder form of Becker muscular dystrophy (BMD) [109]. Hence, restoration of the dystrophin reading frame for a frame-shifting deletion or by-passing a premature termination of translation mutation within an in-frame exon should result in a protein that retains some function. Similarly, it will be imperative to thoroughly investigate which *DYSF* exons other than exon 32 are dispensable, and therefore amenable to AO-mediated exon skipping. Non-essential exons carrying nonsense, missense, or frame-shifting indel mutations may be removed by steric-blocking AOs to yield isoforms that retain some dysferlin function. Similarly, cryptic exons and some splicing mutations could be addressed as was achieved with *Milasen* to restore normal splicing.

#### 3.5.2. Overcoming Limitations and Advancing Delivery Strategies of AO

Antisense therapy presents a promising and potentially adaptable approach to treating a variety of disorders. However, this therapeutic class has some limitations that necessitate careful consideration to maximize benefits while minimizing risks and challenges. These limitations include restricted tissue penetration, off-target effects, limited duration of action, potential immune activation, high cost, and delivery challenges [110].

Chemical modifications are essential for improving the delivery, efficiency, stability, and target specificity of AOs. Altering the phosphate backbone of AOs can enhance their stability and resistance to nuclease degradation. Phosphorothioate (PS) backbone modification, where a sulfur atom replaces one of the non-bridging oxygen atoms in the phosphate backbone, is one of the most commonly used modifications for this purpose. Modifying the sugar moiety of nucleotides by incorporating 2′-O-methyl (2′-OMe), 2′-O-methoxyethyl (2′-MOE) or 2′-fluoro (2′-F) modifications, can further improve the stability of AOs while maintaining their RNA binding affinity [111,112]. Substituting specific nucleotide bases with modified analogs, such as locked nucleic acid (LNA) or peptide nucleic acid (PNA) can also enhance the binding affinity and specificity of AOs to their target RNA sequences [94,113,114]. Phosphorodiamidate morpholino oligomers (PMO) replace the deoxyribose/ribose moiety with a morpholine ring and substitute the charged phosphodiester inter-subunit linkage with an uncharged phosphorodiamidate linkage. This alteration renders PMOs nuclease-resistant and charge-neutral [115]. Another variation of the morpholino oligomers are thiomorpholino oligonucleotides (TMOs), a recently developed novel nucleic acid analog consisting of a morpholino nucleoside connected by thiophosphoramidate internucleotide linkages [116]. Several studies are currently underway to evaluate TMO chemistry in vitro and in vivo [116,117,118].

Conjugating AOs with lipophilic moieties, such as cholesterol or fatty acids, can promote their cellular uptake and intracellular delivery by facilitating their interaction with cell membranes and endosomal escape [119]. In addition, conjugating AOs with cell-penetrating peptides (CPPs) or other cell-targeting ligands can enhance their cellular uptake and tissue-specific delivery by promoting receptor-mediated endocytosis or translocation across cell membranes [120,121,122]. The safety profile of the neutrally charged PMOs could be due in part to the relatively poor cellular uptake, an issue that is being addressed by conjugation to cell-penetrating peptides [123,124]. An ongoing study showed that SRP-5051 (a PMO targeting DMD exon 51 conjugated to a proprietary cell-penetrating peptide) could offer greater efficacy with less frequent dosing than *Exondys* 51, the FDA-approved PMO targeting exon 51 for the most common subset of Duchenne MD patients. Sarepta’s predictive model shows that SRP-5051 at 30 mg/Kg after 12 weeks dosed monthly resulted in 18 times higher exon skipping and eight times higher dystrophin production compared to *Exondys* 51 that is administered weekly at 30 mg/Kg [125]. Treatment with SRP-5051 greatly reduces the impost on patients; the frequency of clinic visits is reduced, and a lower dosage is required while generating more of the induced dystrophin isoform. Encapsulating AOs within nanoparticles, such as lipid nanoparticles (LNPs) [126,127,128] or polymeric nanoparticles [129,130], can protect them from degradation, hence enhancing their circulation time, and facilitate their targeted delivery to specific tissues or cells.

## 4. Conclusions

As is the case with many inherited diseases, LGMD2B is currently considered to be an untreatable neuromuscular condition with poor prognosis [131]. Several researchers are studying potential treatments, and few have reached the clinical trial stage. Multiple therapeutic approaches including gene replacement, CRISPR/Cas9 gene editing, readthrough strategies, small molecule modulators of pathology, and AO-mediated exon skipping are being explored as potential treatments for LGMD2B. As with all ongoing research, there is an expectation that forthcoming treatments will greatly enhance quality of life for individuals with dysferlinopathy. However, more studies are required to better assess the novel therapeutic approaches to verify both efficacy and safety. The trajectory of dysferlinopathy treatment hinges on integrated research, novel therapeutic approaches, and cooperative endeavors that prioritize patient welfare and enhances the quality of life.
